# The usefulness of MDCT-myelography for patients with spontaneous intracranial hypotension

**DOI:** 10.1186/s40064-016-2060-5

**Published:** 2016-04-11

**Authors:** Seung Woo Choi, Joong Mo Ahn, Joon Woo Lee, Kyung Seok Park, Heung Sik Kang

**Affiliations:** Department of Radiology, Seoul National University Bundang Hospital, 300 Gumi-dong, Bundang-gu, Seongnam, Gyeongi-do 13620 South Korea; Department of Neurology, Seoul National University Bundang Hospital, Seongnam, Gyeongi-do 13620 South Korea

**Keywords:** Multidetector CT-myelography, CSF leakage, Spontaneous intracranial hypotension

## Abstract

**Purpose:**

The detection and localization of cerebrospinal fluid (CSF) leakage in patients with spontaneous intracranial hypotension (SIH) is important. The aim of this study was to evaluate the usefulness of multidetector CT (MDCT)-myelography in patients with SIH.

**Methods:**

A radiologist retrospectively searched the electronic database to find the patients who had undergone MDCT-myelography for SIH between October 2010 and September 2014. In the MDCT-myelographic treatment, the patient’s whole spine was scanned by 64 or 256 channel MDCT scanners after being injected with 20 ml of contrast agent via L3/4 interlaminar space under fluoroscopic guidance. Three radiologists in consensus determined the presence, pattern (“pseudodiverticular sign” or “gray-rim sign”) and level of contrast leakage outside the dural sac.

**Results:**

Eighteen patients (M:F = 9:9; mean age, 36.2; age range 17–56) were finally included in this study. CSF leakage was detected in 17 of 18 patients. CSF leakage pattern was described as a “pseudodiverticular sign” in five and “gray-rim sign” in ten patients. Leakage level could be determined in ten patients based on MDCT-myelography.

**Conclusions:**

MDCT-myelography was useful to detect CSF leakage and to guess the leakage level in patients with SIH.

## Background

Spontaneous intracranial hypotension (SIH) is difficult to diagnose with clinical spectrum or imaging findings (Park and Kim 2009). In some studies, it was found that there were at least a few cases satisfying the International Classification of Headache Disorders (ICHD-2) criteria for SIH with typical imaging features (Schievink et al. 2008, 2011).

SIH is usually caused by cerebrospinal fluid (CSF) leakage through the spinal dural sac. At present, it is known that targeted blood patch (Cho et al. 2011; Waguri et al. 2002; Kranz et al. 2011) can be effective in the treatment of SIH, so being able to identify the precise localization of the CSF leakage site is important for more effective treatment.

Several dynamic studies for localization of CSF leakage such as spinal MRI, radioisotope cisternography, and CT-myelography have been attempted.

In our institute, we have, for the last few years, used multidetector CT (MDCT) scanning after myelography (named as “MDCT-myelography”) to localize the CSF leakage site and experienced that MDCT-myelography is a very valuable method to localize CSF leakage sites. However, to the best of our knowledge, there have been no reports about the usefulness of MDCT-myelography to localize the CSF leakage site. In addition, we noticed an interesting finding as a sign of CSF leakage in axial CT images, which was named “gray-rim sign.” “Gray-rim sign” was defined as occurring if a less high-attenuated rim due to leaked contrast was seen around densely high-attenuated contrast inside the dural sac on axial CT images.

The aim of this study was to evaluate the usefulness of MDCT-myelography to detect CSF leakage sites in patients with clinically suspected SIH, and to evaluate the incidence of “gray-rim sign.”

## Methods

### Patient selection

This study was approved by institutional review board, and informed consent was waived. In our institute, MDCT-myelography is considered for patients who are (1) diagnosed with SIH by clinical symptom and typical brain MR findings, and (2) have intractable SIH after conservative management such as hydration, bed rest for more than 1 week. Inclusion criteria were patients who were clinically suspected of SIH and had undergone MDCT-myelography for it. A radiologist retrospectively searched our electronic database to find those patients between October 2010 and September 2014. Patients who had undergone MDCT-myelography for postoperative or traumatic condition were excluded.

### Technique of MDCT-myelography

Lumbar puncture procedures were performed by using a 22-gauge needle at the subarachnoid space via L3/4 or L4/5 interlaminar approach under fluoroscopic guidance by one of four spine radiologists and myelography was done by slowly injecting 20 ml of contrast agent (Omnipaque 300 mg I/ml, Iohexol, GE Healthcare, Piscataway, NJ). During injection of the contrast agent, spot images were obtained for the whole spine from lumbar to cervical area to find any leakage site.

After myelography, the whole spine was scanned by 64- or 256-channel MDCT (I; BRILIANCE 64, PHILIPS, Cleveland, USA, II; ICT 256, PHILIPS, Cleveland, USA). The scan parameters were as follows: thickness (I, 2.0 mm; II, 1.0 mm), increment (I, 1.0 mm; II, 0.5 mm), collimation (I, 64*0.625; II, 128*0.625), pitch (I, 1.203; II, 0.601), rotation time (I, 1; II, 0.5), matrix (I, 512; II, 512), 140 kV (both), 300 mA (I) and 200 mA (II). Axial, sagittal, and coronal reformat images were obtained.

### Image analysis

**Fig. 1:**
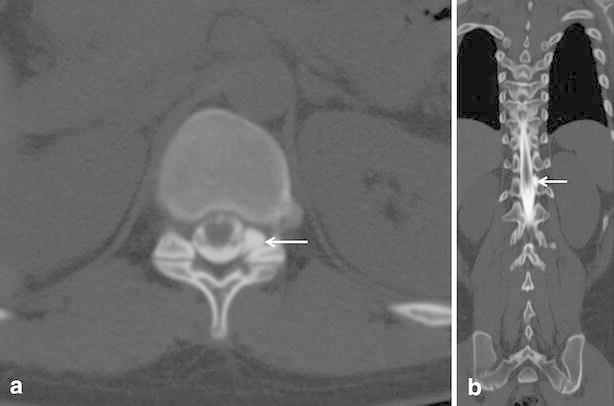
A 17-year-old woman with spontaneous intracranial hypotension (case #1). **a** Axial MDCT-myelography imaging shows focal spillage of contrast media into left foraminal zone at T11/12 level, like diverticulum (“pseudodiverticular sign”). **b** Coronal image also reveals the same finding

**Fig. 2:**
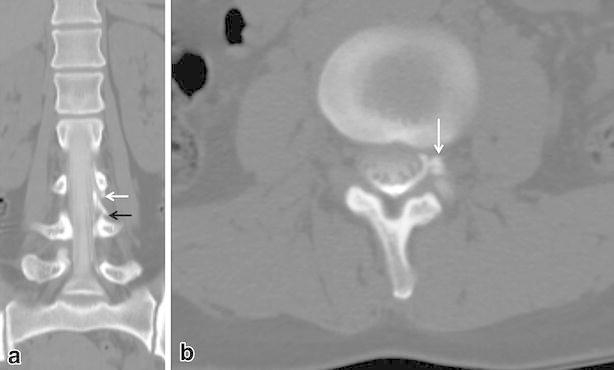
A 37-year-old woman with spontaneous intracranial hypotension (case #5). **a** Coronal MDCT-myelography imaging showed contrast spillage (*black arrow*) below the exiting nerve root sleeve (*white arrow*). **b** Axial image shows contrast leakage like diverticulum (pseudodiverticular sign)

**Fig. 3:**
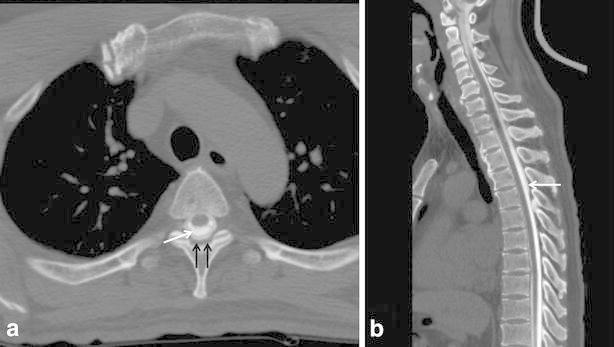
A 56-year-old man with spontaneous intracranial hypotension (case #6). **a** Axial MDCT-myelography imaging shows contrast leakage at the T3 level with “gray-rim sign,” which was defined as high-attenuated peripheral rim due to leaked contrast (*black arrow*) with central densely high-attenuated contrast inside the dural sac (*white arrow*). **b** Sagittal MDCT-myelography shows leakage of contrast media from T1 to T4 level

**Fig. 4:**
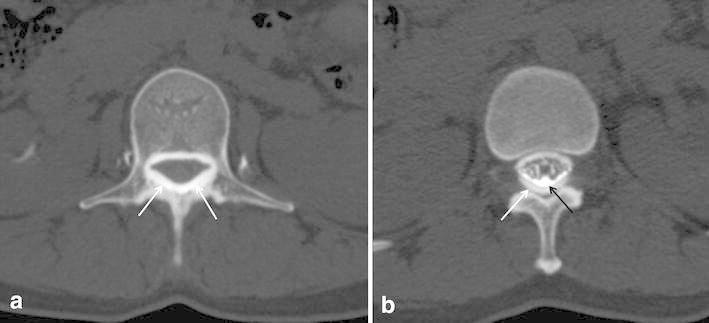
A 37-year-old woman with spontaneous intracranial hypotension (case #2). Initial myelography failed due to extensive subdural contrast injection. Axial MDCT-myelography (**a**) revealed a dense high-attenuated subdural area (*white arrow*) with dark attenuated dural sac. So, blind epidural blood patch was done in the C7/T1 level, but the symptom did not improve after that. One week later MDCT-myelography was repeated. On axial CT image (**b**), contrast leakage was seen at the L1 level with “gray-rim sign” (peripheral leaked contrast with *white arrow* and central dense contrast with *black arrow*). Targeted epidural blood patch was done at the L1 level; the symptoms of the patient relieved dramatically, and she was discharged the next day

The presence of contrast leakage on MDCT-myelography was determined by three radiologists in consensus if contrast agent was seen outside the dural sac. Contrast leakage pattern was evaluated by the three radiologists in consensus: pseudo-diverticular or gray-rim. We defined “pseudodiverticular sign” if there was focal contrast projection outside the dural sac like diverticulum (Figs. 1, 2). “Gray-rim sign” was defined if less high-attenuated rim due to leaked contrast was diffusely seen around densely high-attenuated contrast inside the dural sac on axial CT images (Figs. 3, 4). Based on MDCT-myelography, three spine radiologists determined the leakage level in consensus with the following criteria: (1) the level at which the contrast agent was localized in the epidural space in MDCT-myelography, or, (2) the level at which the contrast leakage was most densely seen and most prominent if the contrast leakage was diffusely seen in the epidural space on MDCT-myelography.

### Clinical outcome after targeted epidural blood patch

Targeted epidural blood patch was done for all patients in the suspected leakage site based on the radiologic report of MDCT-myelography by one of two anesthesiologists. In September 2014, a radiologist retrospectively reviewed the medical chart to evaluate the clinical outcome after targeted epidural blood patch.

### Ethics statement

This retrospective study was approved by the institutional review board of our hospital, and informed consent was waived (Number of IRB; B-1112-142-104).

## Results

Eighteen patients (M:F = 9:9, mean age; 36.2, age range 17–56) were finally included in this study. All patients were suspected of SIH by clinical symptoms and typical brain MR imaging executed by MDCT-myelography. CSF leakage was accurately detected in 17 of these 18 patients (94.4 %). The level of CSF leakage was different (Table 1): six patients at C-spine level, five patients at T-spine level, seven patients at L-spine level, and seven patients were showed extensive CSF leakage at multiple spine levels.

**Table 1:** Summary of multidetector CT-myelography findings of seven patients with diagnosis of spontaneous intracranial hypotension

Patient no.	CSF leakage	Leakage pattern	Location of CSF leakage	Exact location of CSF leakage
1	+	Pseudodiverticular	Left T11/12	+
2	+	Gray rim	L1	+
3	−		−	−
4	+	Gray rim	T12/L1	+
5	+	Pseudodiverticular	Left L2/3 and L3/4	+
6	+	Gray rim	T3	+
7	+	Gray rim	From C5/6 to L1/2	Extensive
8	+	Gray rim	From C1/2 to T10/11	Extensive
9	+	Pseudodiverticular	Right L3/4 and L4/5	+
10	+	Gray rim	L4/5/S1	+
11	+	Gray rim and pseudodiverticular	From T12 to L2	Extensive
12	+	Gray rim	From cervical to upper T-spine	Extensive
13	+	Gray rim	T-spine	Extensive
14	+	Pseudodiverticular	Both T11/12	+
15	+	Gray rim	C5/6/7 and T7/8/9	Extensive
16	+	Pseudodiverticular	Right C5/6	+
17	+	Gray rim	T5/6	+
18	+	Gray rim and pseudodiverticular	From C4 to T4	Extensive

Five patients showed “pseudodiverticular sign” and ten patients showed “gray-rim sign” and two patients showed mixed all signs. For the seven patients with “pseudodiverticular sign,” contrast leakage was focally seen in the neural foraminal zone (Figs. 1, 2). In 12 patients with “gray-rim sign,” diffuse spillage of contrast agent into anterior or posterior epidural space was demonstrated on MDCT-myelography. Among the 18 patients, the leakage sites could be localized in 10 patients. In the remaining patient, the leakage site could not be determined due to the extensive and large amount of leakage. As a result, the leakage site could be found exactly in 10 patients based on MDCT-myelography.

All 17 of the patients underwent targeted epidural blood patch by one of two anesthesiologists for the suspected leakage site based on MDCT-myelography. Fourteen of these 17 patients showed dramatic symptom improvement. Two patients could not recover by 1st attempt of epidural blood patch, and needed repeated epidural blood patch at the same site for the symptom relief. In one patient, initial myelography was failed due to extensive subdural contrast injection, so epidural blood patch was tried for the C7/T1 level. After that, the patient’s symptom did not improve. One week later, after waiting for absorption of previously injected contrast, MDCT-myelography was repeated and revealed a leakage site on the L1 level (Fig. 4). Targeted epidural blood patch was then done for the level, and the patient’s symptoms improved dramatically and she was discharged the next day.

## Discussion

It is well known that SIH has various symptoms and is difficult to diagnose clinically. Therefore, several tools such as conventional myelography, radionuclide cisternography, CT-myelography, and MR-myelography have been suggested for the diagnosis of SIH. Conventional myelography lacks cross-sectional features and anatomic superposition. Radionuclide cisternography is an indirect and direct confirmatory test of SIH. But among the disadvantages of Radionuclide cisternography are its poor spatial resolution, its invasiveness, and the possibility of radioisotope extravasation through the needle tract, which can result in inaccuracy in its interpretation.

CT myelography is considered the most reliable imaging technique for localizing the actual site or spinal level of a CSF leakage (Patrick and Bahram 2003; Fujimaki et al. 2002). However, the rate of identification of the spinal level of a CSF leakage still does not come up to the expectations of clinicians. Plus, CT myelography has a risk related to radiation and may be time-intensive especially in multiple leakage site of CSF. Dynamic CT myelography is a good technique for detecting the site of a CSF leakage especially in case with meningeal diverticula, however, this technique would not be used initially and may have a limitation in diagnosis of extensive CSF leakage (Patrick and Bahram 2003; Kumar et al. 2015) due to relatively small field of view.

MR-myelography provides multiplanar capabilities without risk of radiation exposure and is an excellent approach to depict the anatomy of CSF spaces (Selcuk et al. 2010; Albayram et al. 2008; Watanabe et al. 2009; Wang et al. 2009). But also, there is a relatively low sensitivity compared to CT myelography.

Recently, intrathecal gadolinium MR myelography has been described as a well-tolerated and sensitive examination in the evaluation of CSF leakage, compared with conventional CT myelography (Chazen et al. 2014). However, that has a limitation in detection of multiple leakage sites because of long scan time needed for large field of view.

Accordingly, it has been most important to guess at the presence of a CSF leakage site not only to find CSF leakage but in order to determine the need for the widely used targeted epidural blood patch for the treatment of SIH (Kranz et al. 2011).

In our study, the leakage site was identified by MDCT-myelography. The advantages of MDCT-myelography are as follows: it enables that whole spine level is scanned at one time rapidly, and suggest coronal and sagittal images by reconstruction. Of our patients, 17 were found to have CSF leakages and recovered by epidural blood patch.

The level of CSF leakage was various, as was the leakage pattern. In some cases, it would have been difficult to distinguish CSF leakage from contrast filling on the root sleeve although thin cut axial images. Coronal images were found to be useful for the diagnosis as the contrast spillage was identified before it reached to the next level after passing the root sleeve on the coronal image (Fig. 2).

Five of the cases of CSF leakage were focally seen as “diverticulum”. In ten cases, we could see the less high-attenuated contrast agent outside the dural sac, so we identified it as “gray-rim sign”. We suggest that “pseudodiverticular sign” or “gray-rim sign” are pathognomic findings for CSF leakage on MDCT-myelography.

To select the site of epidural blood patch, it is not mandatory to search obsessively for the correct leakage site itself. We think that localization of a prominent leakage site is sufficient to do an epidural blood patch, for example, upper thoracic, cervicothoracic, thoracolumbar, lumbar, etc. So, in most cases of SIH, MDCT-myelography can play an important role not only by detecting the leakage site itself, but also by implying the most prominent leakage levels.

Interestingly, in one patient (Fig. 4), we made a mistake during myelography. We injected contrast agent into the subdural space, which we were able to detect on MDCT-myelography. So, an anestheologist tried to apply an epidural blood patch on C7/T1 by clinical decision, because patients complained of shoulder and neck pain too. However, there was no symptom improvement. A repeat of MDCT-myelography 1 week later detected the leakage site and determined that the targeted epidural blood patch had been successfully done. By this case, we learned the two points of the following messages. Firstly, because CSF pressure is usually very low in patients with SIH, a dural puncture is not made so easily and subdural or epidural contrast injection is possible during myelography. If subdural or epidural contrast injection is made for MDCT-myelography, we cannot determine the CSF leakage. Secondly, targeted epidural blood patch is very effective for management of patients with SIH.

This study was limited as it was conducted as a retrospective study. Also, the size of the sample was small because we considered MDCT-myelography only in patients who did not respond to conservative therapy. It is not feasible to conduct a prospective study because SIH is a rare condition. However, this is a preliminary study to introduce the usefulness of MDCT-myelography to evaluate SIH patients.

In conclusion, MDCT-myelography facilitates a more accurate detection of CSF leakage sites in SIH patients and “gray-rim sign” is common on axial CT images.
